# Divergent selection for natural antibodies in poultry in the presence of a major gene

**DOI:** 10.1186/s12711-022-00715-9

**Published:** 2022-03-21

**Authors:** Henk Bovenhuis, Tom V. L. Berghof, Marleen H. P. W. Visker, Joop A. J. Arts, Jeroen Visscher, Jan J. van der Poel, Henk K. Parmentier

**Affiliations:** 1 Animal Breeding and Genomics Centre, Department of Animal Sciences, Wageningen University, Wageningen, The Netherlands; 2 Adaptation Physiology Group, Department of Animal Sciences, Wageningen University, Wageningen, The Netherlands; 3 Hendrix Genetics Research Technology & Service B.V, P.O. Box 114, 5830 AC Boxmeer, The Netherlands; 4 Reproductive Biotechnology, TUM School of Life Sciences, Technical University of Munich, Liesel-Beckmann-Strasse 1, 85354 Freising, Germany

## Abstract

**Background:**

Natural antibodies (NAb) are antibodies that are present in a healthy individual without requiring previous exposure to an exogenous antigen. Selection for high NAb levels might contribute to improved general disease resistance. Our aim was to analyse the genetic background of NAb based on a divergent selection experiment in poultry, and in particular the effect of a polymorphism in the *TLR1A* gene.

**Methods:**

The study population consisted of a base population from a commercial pure-bred elite white leghorn layer line and seven generations of birds from a High and Low selection line. Birds were selected for total KLH-binding NAb titer (IgTotal). An enzyme-linked immunosorbent assay was performed to determine NAb titers in blood plasma for IgTotal and the antibody isotypes IgM and IgG. NAb titers were available for 10,878 birds. Genotypes for a polymorphism in *TLR1A* were determined for chickens in generations 5, 6 and 7. The data were analysed using mixed linear animal models.

**Results:**

The heritability estimate for IgM was 0.30 and higher than that for IgG and IgTotal (0.12). Maternal environmental effects explained 2 to 3% of the phenotypic variation in NAb. Selection for IgTotal resulted in a genetic difference between the High and Low line of 2.4 titer points (5.1 genetic standard deviation) in generation 7. For IgM, the selection response was asymmetrical and higher in the Low than the High line. The frequency of the *TLR1A C* allele was 0.45 in the base population and 0.66 and 0.04 in generation 7 of the High and Low line, respectively. The *TLR1A* polymorphism had large and significant effects on IgTotal and IgM. Estimated genotypic effects suggest full dominance of the *TLR1A C* allele. Significant *TLR1A* by generation interactions were detected for IgM and IgTotal.

**Conclusions:**

The effect of a polymorphism in the *TLR1A* gene on IgTotal and IgM NAb was confirmed. Furthermore, we provide experimental verification of changes in allele frequencies at a major gene with dominant gene action on a quantitative trait that is subjected to mass selection. *TLR1A* by generation interactions indicate sensitivity to environmental factors.

**Supplementary Information:**

The online version contains supplementary material available at 10.1186/s12711-022-00715-9.

## Background

Diseases pose serious and increasing challenges to livestock production. Diseases not only affect production efficiency, and thus environmental impact, but also consumers’ attitude towards food products of animal origin. In recent years, poultry layer production has shifted in several countries from battery cages to free roaming housing systems. This has increased contact between chickens, resulting in a higher risk of pathogen spreading [1, 2]. Another recent trend is the restricted use of antibiotics in order to reduce the risk of antibiotic resistance [1]. These developments require other ways to limit the impact of diseases on poultry production. Selective breeding could provide an interesting option, as part of an integrated animal health management approach.

There are several aspects that complicate selective breeding for disease resistance. First, elite breeding animals are usually kept under strict hygienic conditions and, as a result an animal’s ability to fight pathogens is not or poorly expressed. Second, traits that capture differences in disease resistance between animals must be identified. Selection should preferably not be for resistance to one or a few diseases but for enhanced immune competence that results in a broad (general) disease resistance. Furthermore, traits that reflect general disease resistance should be easy and cheap to measure and ideally early in the animal’s life.

Natural antibodies (NAb) could be of value in selection for general disease resistance. NAb are antibodies that are present in a healthy individual without requiring previous exposure to an exogenous antigen [3]. NAb are regarded as a humoral part of the innate immunity, show a broad specificity repertoire, and act as a first line of defence against infections. Furthermore, NAb can be measured relatively easily in blood samples and at a young age. In several animal species, higher NAb levels have been associated with better health or increased disease resistance [4–7]. In laying hens, high levels of NAb have been related to a higher probability of survival during the laying period [8–10]. Furthermore, we showed in a previous study that selection for high NAb against keyhole limpet hemocyanin (KLH) leads to higher survival of chickens that are challenged with avian pathogenic *Escherichia coli* [11]. Therefore, selection for high NAb levels might contribute to improved general disease resistance in poultry.

In previous studies on poultry, we showed that NAb against KLH are heritable [12, 13] and affected by maternal environmental effects [14]. By performing a genome-wide association study (GWAS), we identified a genomic region on chicken chromosome 4 with a major effect on IgM NAb [15]. This chromosomal region contains the *toll-like receptor 1 family member A* (*TLR1A*) gene, in which a polymorphism was identified as the most likely causal variant affecting the level of NAb [15]. Studies by Berghof et al. [14, 15] were based on a population that formed the basis of a divergent selection experiment that was used for the current study. In addition to information from the base population, data were available from seven generations of mass selection for high and low total KLH binding NAb titers. Chickens in generations 5, 6 and 7 of the selection experiment were also genotyped for the polymorphism identified in the *TLR1A* gene. Using this large data set, we performed a detailed study of the genetic effects (direct and maternal genetic), maternal environmental effects, sex effects on NAb, and specifically the role of the *TLR1A* polymorphism. Furthermore, the availability of *TLR1A* genotypes provided a unique opportunity to quantify the effect of mass selection on allele frequencies at a major gene for NAb titers.

## Methods

### Experimental design

The birds used in this study were from a commercial pure-bred elite white leghorn layer line provided by Hendrix Genetics (Boxmeer, The Netherlands). The study population consisted of a base population of birds from the same layer line, that had not been selected for NAb, and seven subsequent generations that were divergently selected for total KLH-binding NAb titer (IgTotal), i.e. from High and Low selection lines.

The base population consisted of two batches of chickens. The first batch was previously described and used in studies by van der Klein et al. [16] and Berghof et al. [14] to estimate genetic parameters for NAb traits. The second batch was used in a GWAS that was described in Berghof et al. [15]. In total, 437 chickens overlapped between the two batches and were included in both studies reported in [14] and [15]. Combined, 4855 chickens (3099 females and 1756 males) were available and are referred to as the base population. All birds were maintained at the nucleus breeding facilities of Hendrix Genetics. Birds from the first batch were divergently selected for IgTotal NAb: 24 males and 44 females with the highest NAb titers were selected as parents for the High line and 24 males and 48 females with the lowest NAb titers were selected as parents for the Low line (i.e. mass selection). Our aim was to mate one male to two females and one female to one male.

Incubation of eggs and housing of chickens for the selection lines (from generation 1 onwards) was at the research facility “Carus” from Wageningen University & Research, according to standard production practices. In each generation, our aim was to select 25 males and 50 females per line, but for some generations the number of selected parents deviated slightly (see Additional file 1: Table S1). Selection of parents was as described for the base population and was done within lines. Our aim was to mate each male to two females, avoiding full or half sib matings. All birds from both lines of a given generation were raised in a single batch. The number of birds per generation ranged from 526 to 1135 and the selection experiment included 6026 birds (~ 50% males and ~ 50% females) with NAb antibody titers (see Table 1).

**Table 1:** Descriptive statistics (averages, with standard deviations in parentheses) of total (IgTotal), IgM, and IgG KLH-binding natural antibody titers in a White Leghorn chicken population divergently selected for IgTotal, resulting in a High and a Low line

Generation	IgM			IgG			IgTotal			N^a^
	Low	High	H-L^b^	Low	High	H-L^b^	Low	High	H-L^b^	
Base	7.10_(1.43)_		0.00	6.16_(1.57)_		0.00	6.89_(1.51)_		0.00	4855
G1	6.11_(0.97)_	6.61_(1.01)_	0.50	5.81_(1.37)_	6.27_(1.28)_	0.46	5.89_(1.33)_	6.38_(1.21)_	0.49	946
G2	5.75_(0.91)_	6.68_(0.85)_	0.93	5.17_(1.37)_	5.99_(1.34)_	0.82	5.17_(1.39)_	6.36_(1.28)_	1.19	820
G3	5.21_(0.98)_	6.40_(0.84)_	1.19	4.96_(1.78)_	5.84_(1.49)_	0.88	6.77_(1.84)_	8.11_(1.60)_	1.34	526
G4	4.33_(1.10)_	6.11_(0.91)_	1.78	4.39_(1.60)_	6.03_(1.37)_	1.64	4.26_(1.58)_	6.36_(1.29)_	2.10	915
G5	4.90_(1.20)_	6.69_(0.88)_	1.79	4.76_(1.66)_	6.52_(1.43)_	1.76	4.57_(1.72)_	6.59_(1.30)_	2.02	1135
G6	3.61_(1.16)_	6.32_(1.02)_	2.71	4.41_(1.88)_	6.70_(1.55)_	2.29	5.50_(1.40)_	7.50_(0.95)_	2.00	707
G7	3.62_(1.44)_	6.55_(0.99)_	2.93	3.77_(1.49)_	6.14_(1.01)_	2.37	3.32_(1.45)_	5.96_(1.02)_	2.64	974
Total										10,878

^a^Number of observations for IgTotal; the numbers of records for IgM and IgG differ slightly from those for IgTotal

^b^All differences between the High and Low selection lines are significant from G1 onwards, when tested based on a two-sided t-test

### NAb phenotypes

In the base population, blood plasma samples were taken at 15 and 19 weeks of age for males and females, respectively, following routine blood sampling procedures at Hendrix Genetics. In generations 1 to 7, blood plasma samples were taken at 16 weeks of age for both males and females. Plasma samples were collected and stored at − 20 °C until their use to measure IgTotal, IgM, and IgG binding KLH. An indirect two-step enzyme-linked immunoassay (ELISA) was used, as described by Berghof et al. [14]. For this purpose, 96-well plates were coated with 2 µg/mL KLH in 100 µL coating buffer. Tested plasma dilutions were 1:40, 1:160, 1:640 and 1:2560. A duplicate standard positive was incorporated on each ELISA plate. Plates were incubated and washed, and then incubated with different polyclonal antibodies: 1:20,000-diluted rabbit-anti-chicken IgG heavy and light chain (IgTotal), 1:20,000-diluted goat-anti-chicken IgM, or 1:40,000-diluted goat-anti-chicken IgG, which were all labelled with horse radish peroxidase. Binding of the antibodies to KLH was visualized by adding 100 µL of substrate buffer. The reaction was stopped after 15 min by adding 50 µL of 1.25 M H_2_SO_4_. Extinctions were measured at 450 nm. Antibody titers were calculated as log_2_ values of the dilutions that gave an extinction that was closest to 50% of E_MAX_, where E_MAX_ represents the mean of the highest extinction of the standard positive plasma samples.

For the base population, samples from males and females were analysed on different ELISA plates [14]. For generations 1 to 7, each plate included samples of both sexes from the High and Low lines. The average number of ELISA plates per antibody isotype and generation was 48.

### TLR1A genotypes

A genomic region on chromosome 4 that is known to be significantly associated with NAb titers contains the *toll-like receptor 1 family member A* (*TLR1A*) gene, which harbors a single nucleotide polymorphism (SNP) that has been identified as the most likely causal variant [15]. This SNP consists of a cytosine (C)/guanine (G) polymorphism and results in a phenylalanine (F)/leucine (L) amino acid substitution in the TLR1A protein at position 126. Imputed genotypes for this *TLR1A* SNP, based on whole genome sequence data, were available for the base population and the frequency of the imputed *TLR1A C* allele was 0.45 [15]. The genotypes at this *TLR1A* SNP for chickens in generations 5, 6 and 7 were determined based on a TaqMan assay. For this purpose, DNA was isolated from blood samples, and 4 µL of the normalized DNA sample was mixed with 8 µL of TaqMan mix that included primers and probes for the SNP. The FAM and VIC fluorescent dyes were used for the *G* and *C* allele probes, respectively. Polymerase chain reactions (PCR) were run for 40 cycles at 95 °C for 15 s and at 60 °C for 1 min. Genotypes were called using the QuantStudio™ Design and Analysis Software v1.4.3 (Thermo Fisher Scientific Corporation). Samples with a high FAM signal were classified as homozygous *G*, those with a high VIC signal as homozygous *C*, and samples with an approximately equal signal for both FAM and VIC were classified as heterozygous. When birds could not be unequivocally assigned to one of the genotypic classes their genotype was set to missing.

### Statistical analyses

In a previous study, we identified significant maternal environmental effects for IgM NAb titers, but not for IgTotal and IgG, and found no evidence for maternal genetic effects [14]. The availability of a large data set, consisting of multiple generations, enhanced the possibilities to disentangle the maternal environmental and (maternal) genetic factors. For this purpose, we analyzed the data using several mixed linear models. All statistical analyses were performed using ASREML 4 [17].

The first model contained a random maternal (environmental) effect and a random additive genetic effect:

1 $${\mathrm{y}}_{\mathrm{ijkl}}=\upmu +{\mathrm{Plate}}_{\mathrm{i}}+{\mathrm{Sex}}_{\mathrm{j}}+{\mathrm{a}}_{\mathrm{k}}+{\mathrm{d}}_{\mathrm{l}}+{\mathrm{e}}_{\mathrm{ijkl}},$$

where ${\mathrm{y}}_{\mathrm{ijkl}}$ is the IgTotal, IgM, or IgG titer, $\upmu$ is the mean, ${\mathrm{Plate}}_{\mathrm{i}}$ is the fixed effect of the ELISA plate (with $\mathrm{i}$ = 1 to 592), ${\mathrm{Sex}}_{\mathrm{j}}$ is the fixed effect of sex (with $\mathrm{j}$ = 1,2; male or female), ${\mathrm{a}}_{\mathrm{k}}$ is the random additive genetic effect of the $\mathrm{k}$th animal, which was assumed to be distributed as $N\left(\boldsymbol{0},\mathbf{A}{\sigma }_{A}^{2}\right)$, where $\mathbf{A}$ is the pedigree-based relationship matrix and ${\sigma }_{A}^{2}$ is the additive genetic variance, ${\mathrm{d}}_{\mathrm{l}}$ is the random effect of the $\mathrm{l}$th dam, which is assumed to be distributed as $N\left(\boldsymbol{0},\mathbf{I}{\sigma }_{m}^{2}\right)$, where $\mathbf{I}$ is the identity matrix and ${\sigma }_{m}^{2}$ is the maternal environmental variance, and ${\mathrm{e}}_{\mathrm{ijkl}}$ is the random residual term, which was assumed to be distributed as $N\left(\boldsymbol{0},\mathbf{I}{\sigma }_{e}^{2}\right)$, where ${\sigma }_{e}^{2}$ is the residual variance. To build the $\mathbf{A}$ matrix, pedigree information of 12,443 individuals from 14 generations was used. Generation effects are confounded with ELISA plate effects and therefore “Generation” was not included as a separate effect in the model. Thus, plate effects not only represent the effect of ELISA plate but also include generation or batch effects.

Heritability and maternal environmental effects were estimated as:

$${h}^{2}=\frac{{\sigma }_{A}^{2}}{{\sigma }_{A}^{2}+{\sigma }_{m}^{2}+{\sigma }_{e}^{2}}$$

$$\mathrm{and}\, {m}^{2}=\frac{{\sigma }_{m}^{2}}{{\sigma }_{A}^{2}+{\sigma }_{m}^{2}+{\sigma }_{e}^{2}}.$$

To test if the maternal environmental effects affected NAb titers significantly, the data were also analysed using a model without a random dam effect:

2 $${\mathrm{y}}_{\mathrm{ijk}}=\upmu +{\mathrm{Plate}}_{\mathrm{i}}+{\mathrm{Sex}}_{\mathrm{j}}+{\mathrm{a}}_{\mathrm{k}}+{\mathrm{e}}_{\mathrm{ijkl}},$$

where effects are as described for Model (1). The likelihood ratio test was used to test for significance of maternal environmental effects:

3 $$LR=2\left[L\left(\widehat{\theta }\right)-L\left({\theta }_{0}\right)\right],$$

where $L\left(\widehat{\theta }\right)$ is the log-likelihood based on Model (1) and $L\left({\theta }_{0}\right)$ is the log-likelihood based on Model (2), i.e. where the variance of maternal environmental effects was restricted to 0. It was assumed that the likelihood ratio test followed a chi-square distribution with one degree of freedom.

To investigate if in addition to maternal environmental effects, maternal genetic effects play a role, the data were analysed using the following model:

4 $${\mathrm{y}}_{\mathrm{ijklm}}=\upmu +{\mathrm{Plate}}_{\mathrm{i}}+{\mathrm{Sex}}_{\mathrm{j}}+{\mathrm{a}}_{\mathrm{k}}+{\mathrm{d}\_\mathrm{e}}_{\mathrm{l}}+{\mathrm{d}\_\mathrm{g}}_{\mathrm{m}}+{\mathrm{e}}_{\mathrm{ijklm}},$$

where effects are as described for Model (1) and ${\mathrm{d}\_\mathrm{e}}_{\mathrm{l}}$ and ${\mathrm{d}\_\mathrm{g}}_{\mathrm{m}}$ are the random maternal environmental and genetic effects, respectively, of the $\mathrm{l}$^th^ dam and $\mathrm{m}$^th^ dam. The assumed (co)variance structure of the random effects was:

$$Var\left(\begin{array}{c}\mathbf{a}\\ \mathbf{d}\_\mathbf{g}\\ \mathbf{d}\_\mathbf{e}\\ \mathbf{e}\end{array}\right)= \left(\begin{array}{cccc}\mathbf{A}{\sigma }_{a}^{2}& \mathbf{A}{\sigma }_{a,m\_g}& \boldsymbol{0}& \boldsymbol{0}\\ \mathbf{A}{\sigma }_{a,m\_g}& \mathbf{A}{\sigma }_{m\_g}^{2}& \boldsymbol{0}& \boldsymbol{0}\\ \boldsymbol{0}& \boldsymbol{0}& \mathbf{I}{\sigma }_{m}^{2}& \boldsymbol{0}\\ \boldsymbol{0}& \boldsymbol{0}& \boldsymbol{0}& \mathbf{I}{\sigma }_{e}^{2}\end{array}\right).$$

The likelihood ratio test (Eq. (3)) was used to test for significance of maternal genetic effects, where $L\left(\widehat{\theta }\right)$ is the log-likelihood based on Model (4) and $L\left({\theta }_{0}\right)$ is the log-likelihood on Model (1). It was assumed that the likelihood ratio test followed a chi-square distribution with two degrees of freedom.

Response to selection was estimated by averaging the estimated breeding values obtained from Model (1) of all individuals in each line-by-generation combination.

To estimate genetic and phenotypic correlations between the three NAb traits, bivariate analyses were performed. Furthermore, to determine if the genetic background of the NAb traits differed between the sexes, titers of males and females were analysed as different traits in bivariate analyses. The bivariate models used were as described for Model (1) but without the effect of sex. The likelihood ratio test (Eq. (3)) was used to test if the genetic correlations differed from 1 by comparing the likelihood of the unrestricted bivariate model with the likelihood of the bivariate model in which the genetic correlation was fixed at 0.999.

To estimate the effect of the *TLR1A* polymorphism, phenotypes and *TLR1A* genotypes from chickens in generations 5 (n = 1105), 6 (n = 681) and 7 (n = 978) of the selection experiment were used in the following model:

5 $${\mathrm{y}}_{\mathrm{ijklmn}} = \upmu +{\mathrm{Plate}}_{\mathrm{i}}+{\mathrm{Sex}}_{\mathrm{j}}+{\mathrm{Line}}_{\mathrm{k}}+{\mathrm{TLR}1\mathrm{A}}_{\mathrm{l}}+{\mathrm{a}}_{\mathrm{m}}+{\mathrm{d}}_{\mathrm{n}}+{\mathrm{e}}_{\mathrm{ijklmn}},$$

where effects are as described for Model (1), ${\mathrm{Line}}_{\mathrm{k}}$ is the fixed effect of line to account for genetic differences between lines. (with $\mathrm{k}$ = 1, 2 for High and Low), and $\mathrm{TLR}1\mathrm{A}$ is the effect of the *TLR1A* SNP (with $\mathrm{l}$ = 1, 2, 3 for genotypes *CC*, *CG*, or *GG*). Additive genetic and maternal environmental variances in these analyses were fixed at the estimates obtained from Model (1) based on the complete data set. Estimates of *TLR1A* genotypic effects of Model (5) were reparametrized into additive and dominance effects as:

$$Additive=\frac{\left(CC-GG\right)}{2}, \mathrm{and}$$

$$Dominance=CG-\left(\frac{CC+GG}{2}\right).$$

Significance of additive and dominance effects was tested using the !CONTRAST statement in ASREML 4 [17]. Additional analyses were performed to test for possible interactions between *TLR1A* and sex, *TLR1A* and Line, *TLR1A* and Generation by adding the respective interactions in Model (5).

## Results

Natural antibody titers for IgTotal, IgM and IgG for 10,878 chickens were available for the current study. A little less than half of the observations were from chickens in the base population. Average NAb titers for the High and Low lines and each generation are in Table 1. NAb titers showed considerable fluctuations from one generation to the next but the difference between the High and Low lines gradually increased over generations, not only for the selection criterion, IgTotal, but also for IgM and IgG; in generation 7, the difference between the High and Low lines was 2.64, 2.93, and 2.37 titer points for IgTotal, IgM, and IgG, respectively.

Remarkably, the standard deviation of the NAb titers was larger in the Low line than in the High line and this difference tended to increase over generations. E.g., in generation 7 the phenotypic standard deviations for IgTotal were 1.45 and 1.02 in the Low and High lines, respectively.

Model comparisons showed that a model with maternal environmental effects (Model (1)) had a significantly better fit than a model without (Model (2)): the likelihood ratio was 26.3 (p < 0.001) for IgTotal, 11.4 (p < 0.001) for IgG, and 25.9 (p < 0.001) for IgM. Adding maternal genetic effects to the model (Model (4)) only resulted in a significant improvement for IgM: the likelihood ratio was 3.2 for IgTotal, 2.2 for IgG, and 6.5 for IgM (p = 0.04). The estimate of the maternal genetic variance for IgM was close to 0 and the estimated genetic correlation between direct and maternal genetic effects was negative and outside the parameter space (− 3.79). Therefore, we concluded that a model with maternal environmental effects (Model (1)) provides the best fit for IgTotal, for IgG, and for IgM. Results for this model are in Table 2.

**Table 2:** Estimated heritabilities and maternal environmental effects (standard errors in parentheses) for IgM, IgG, and total (IgTotal) KLH-binding natural antibody titers

	IgM	IgG	IgTotal
Phenotypic variance (${\sigma }_{p}^{2}$)	1.15_(0.02)_	1.95_(0.03)_	1.85_(0.03)_
Heritability (${h}^{2}$)	0.30_(0.02)_	0.12_(0.01)_	0.12_(0.01)_
Maternal environmental effects (${m}^{2}$)	0.03_(0.01)_	0.02_(0.01)_	0.03_(0.01)_

The heritability estimate for IgM (0.30) was considerably higher than those for IgG and IgTotal (0.12). All three NAb isotypes were significantly affected by maternal environmental effects, which explained 2 to 3% of the phenotypic variation. The ELISA plate effect, which included effects of generation or batch, was highly significant for all three NAb isotypes. When plate was included as a random effect in the model, it explained 22, 18, and 14% of the phenotypic variation for IgT, IgM, and IgG, respectively. Furthermore, there was a significant but relatively small effect of sex on IgM (p = 0.04), but not on IgTotal or IgG. Males had 0.13 titer point lower IgM levels than females.

**Table 3:** Estimated correlations between total (IgTotal), IgM, and IgG KLH-binding natural antibody titers (standard errors in parentheses)

	IgM	IgG	IgTotal
IgM	–	0.77_(0.06)_	0.91_(0.03)_
IgG	0.30_(0.01)_	–	0.94_(0.02)_
IgTotal	0.54_(0.01)_	0.84_(0.003)_	–

Genetic correlations are above the diagonal and phenotypic correlations are below the diagonal

Bivariate analyses showed strong genetic correlation estimates of IgTotal with IgM (0.91) and IgG (0.94) (Table 3). IgG and IgM were also positively correlated but their genetic correlation estimate was lower (0.77). All genetic correlations differed significantly from 1, indicating that these NAb isotypes are partly affected by different genetic effects. The phenotypic correlation of IgG and IgTotal was strong (0.84), whereas that between IgM and IgG was weak (0.30). The correlation between the residuals for IgM and IgG was 0.21 (± 0.01), which indicates that these two NAb titers are largely affected by different random environmental factors. Estimates of the correlation between maternal environmental effects for IgTotal and IgM, of IgTotal with IgG and of IgM with IgG were equal to 0.65 (± 0.10), 0.87 (± 0.05) and 0.41 (± 0.15), respectively.

Table 4 shows the results of the bivariate analyses in which male and female antibody titers were treated as different traits. For IgM, the estimated genetic correlation was 0.96, which is not significantly different from 1. The phenotypic and additive genetic variances for IgM NAb were larger in males than in females. For IgG, the estimated genetic correlation was 0.88, which is significantly different from 1. However, the estimate of the genetic correlation between male and female IgTotal NAb titers was 0.78, which is significantly different from 1 (p = 0.05). Correlations between maternal environmental effects (${\mathrm{r}}_{\mathrm{m}}$) could not be estimated very accurately, with standard errors ranging from 0.23 to 0.42.

**Table 4:** Estimated genetic parameters when male and female total (IgTotal) IgM, and IgG KLH-binding natural antibody titers are treated as genetically different traits in bi-variate analyses (standard errors in parentheses)

	IgM		IgG		IgTotal	
	Male	Female	Male	Female	Male	Female
Phenotypic variance (${\sigma }_{p}^{2}$)	1.28_(0.03)_	1.05_(0.02)_	1.96_(0.04)_	1.93_(0.04)_	1.90_(0.04)_	1.79_(0.03)_
Heritability (${h}^{2}$)	0.30_(0.03)_	0.32_(0.03)_	0.11_(0.02)_	0.14_(0.02)_	0.12_(0.02)_	0.15_(0.02)_
Maternal env. effects (${m}^{2}$)	0.04_(0.01)_	0.04_(0.01)_	0.03_(0.01)_	0.02_(0.01)_	0.05_(0.01)_	0.02_(0.01)_
${\mathrm{r}}_{\mathrm{a}}$	0.96_(0.04)_		0.88_(0.10)_		0.78_(0.11)_	
${\mathrm{r}}_{\mathrm{m}}$	0.74_(0.23)_		0.91_(0.42)_		0.86_(0.34)_	

${\mathrm{r}}_{\mathrm{a}}$ is the correlation between additive genetic effects and ${\mathrm{r}}_{\mathrm{m}}$ is the correlation between maternal environmental effects

**Fig. 1:**
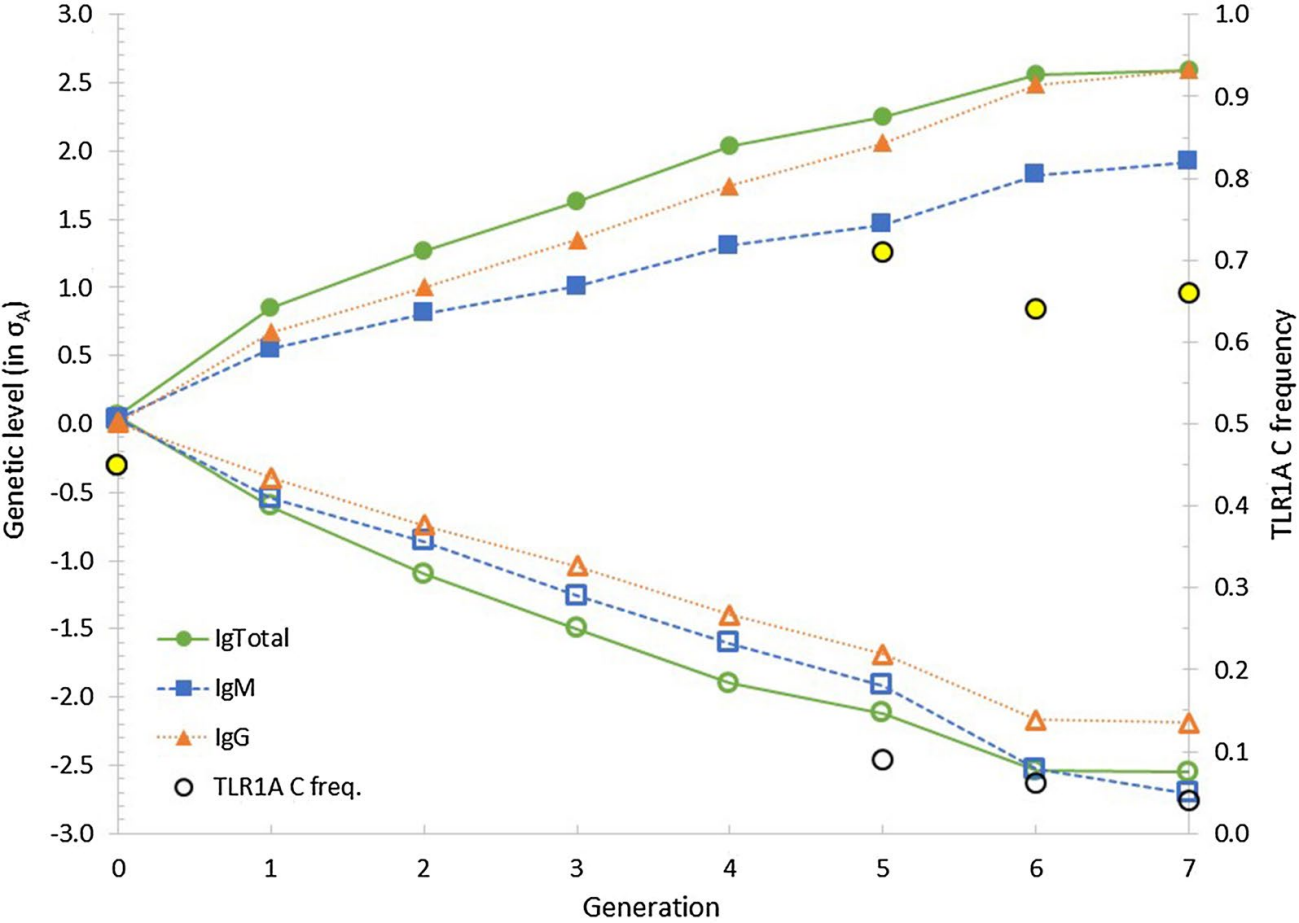
Genetic trend for IgTotal, IgM and IgG natural antibody titers (expressed in genetic standard deviations) and in frequencies of the *TLR1A C* allele for the High (filled marker) and Low lines (open marker) with mass selection on IgTotal. The frequency of *TLR1A C* allele in the base population was based on imputed genotypes, as described by Berghof et al. [15]

Figure 1 shows the genetic trend for NAb titers in the High and Low lines, which was estimated based on a model that accounts for maternal environmental effects (Model (1)). To make the results for IgTotal, IgM and IgG comparable, the genetic trends are expressed in additive genetic standard deviations (${\sigma }_{A}$) (from Table 2). Mass selection for high IgTotal titers increased the genetic levels for IgTotal, IgM, and IgG, whereas the opposite was observed for the Low selection line. In the first round of selection, response was higher due to a higher selection intensity, which could be realized due to a larger number of selection candidates (see Table 1) and (see Additional file 2: Figs. S1 and S2). In the last round of selection, response was lower due to a lower realized selection difference (see Additional file 2: Figs. S1 and S2). Selection for IgTotal titers resulted in a genetic difference of 5.1 ${\sigma }_{A}$ (2.4 titer points) between the High and Low lines. Correlated selection responses for IgM and IgG were 4.6 ${\sigma }_{A}$ and 4.8 ${\sigma }_{A}$, respectively. For IgM, the selection response was asymmetrical, i.e. the genetic level was − 2.7 ${\sigma }_{A}$ and + 1.9 ${\sigma }_{A}$ in the Low and High lines, respectively.

The frequency of the *C* allele was 0.71, 0.64, and 0.66 in generations 5, 6, and 7, respectively, in the High line (Fig. 1), and it was 0.09, 0.06, and 0.04 in generations 5, 6, and 7, respectively, in the Low line. In generation 5 of the Low line, five chickens had the *CC* genotype*,* but in generations 6 and 7 there were no chickens with the *CC* genotype.

Table 5 shows that the *TLR1A* polymorphism had a highly significant effect on IgM and IgTotal NAb titers and a smaller but significant effect on IgG (p = 0.048). The *CC* and *CG* genotypes had more than 1 titer point higher IgM NAb levels than the *GG* genotype. As for IgM, the *CC* and *CG* genotypes had higher IgTotal NAb titers than the *GG* genotype. For IgG, the heterozygous *CG* genotype had higher titers than the *GG* and *CC* genotypes. For IgM and IgTotal, both additive and dominance effects of *TLR1A* were significant. For IgG, the additive effect was not significant, but the dominance effect was (p = 0.015).

**Table 5:** Estimated effects of the *TLR1A* polymorphism on total (IgTotal), IgM, and IgG KLH-binding natural antibody titers based on chickens in generations 5 (n = 1105), 6 (n = 681), and 7 (n = 978) (standard errors in parentheses)

	IgM	IgG	IgTotal
*GG*	0	0	0
*CG*	1.01_(0.07)_	0.22_(0.10)_	0.49_(0.09)_
*CC*	1.12_(0.09)_	0.07_(0.13)_	0.44_(0.11)_
p-value	< 0.001	0.048	< 0.001

For IgG, no significant interactions of the *TLR1A* polymorphism with Sex, Line, or Generation were detected. The *TLR1A* effect on IgM and IgTotal suggested full dominance of the *C* allele and, therefore, only two genotype classes (*GG* and *CG*/*CC*) were distinguished for the interaction analyses. For IgM, a significant (*TLR1A* $\times$ Sex) interaction was detected (p = 0.004). The effect of the *TLR1A* polymorphism (*CC*/*CG* versus *GG*) was larger in males (1.15) than in females (0.92). For IgTotal, neither a significant (*TLR1A* $\times$ Sex) interaction, nor a significant (*TLR1A* $\times$ Line) interaction were detected, which indicates that the *TLR1A* effects did not differ significantly between the High and Low selection lines. For IgM, the (*TLR1A* $\times$ Line) interaction was suggestive (p = 0.051), with the *TLR1A* effect tending to be larger in the Low (1.17) than in the High line (0.89).

Remarkably, highly significant (*TLR1A* $\times$ Generation) interactions were detected, both for IgM (p < 0.001) and IgTotal (p = 0.008). For IgTotal, the difference between the *CC*/*CG* and *GG* genotype classes was 0.43, 0.23, and 0.71 titer points in generations 5, 6, and 7, respectively. For IgM, the difference between the *CC*/*CG* and *GG* genotype classes was 0.70, 1,22, and 1.28 titer points in generations 5, 6, and 7, respectively.

## Discussion

In this paper, we describe a divergent selection experiment for high and low levels of natural antibodies in chickens using mass selection. To enhance immune competence or disease resistance, several selection experiments for single- or multi-trait immune responses have been performed in poultry (reviewed in [18, 19]). These studies demonstrated the feasibility of selecting for immune responses. Heritability estimates for antibody responses in these studies ranged from 0.20 to 0.35 (reviewed in [19]), which is in line with our results for IgM NAb. In some of these selection experiments, differences in major histocompatibility (MHC) allele frequencies were observed between the divergent lines (reviewed in [19]), which might be due to selection.

To our knowledge, our study is the first divergent selection experiment for a non-antigen specific immune trait, i.e. natural antibodies. The large number of phenotypes collected over multiple generations allowed a detailed study of the maternal environmental and genetic factors that affect NAb and, in particular, the effect of a polymorphism in the *TLR1A* gene. Our results provide unique experimental verification of changes in allele frequencies at a major gene with dominant gene action on a quantitative trait that is subjected to mass selection.

### Heritabilities and maternal effects

In a previous study, we estimated genetic parameters for NAb using data on 3689 chickens from the base population [14]. In the current study, we used data from 10,878 chickens from eight generations, which enhanced the ability to disentangle genetic and maternal (genetic) effects. Based on data from the base population, Berghof et al. [14] reported heritability estimates of 0.12 for IgTotal, 0.07 for IgG and 0.14 for IgM, and significant maternal environmental effects for IgM (0.06) but not for IgTotal and IgG. The current study shows that IgTotal, IgM, and IgG are all significantly affected by maternal environmental effects, and to the same extent (${m}_{2}$ = 0.02–0.03). The heritability estimate for IgM in the current study is considerably higher than that previously reported by Berghof et al. [14], i.e. 0.30 (SE = 0.02) versus 0.14 (SE = 0.05). This difference can be explained by the larger number of birds with NAb phenotypes in the current study that were collected over multiple generations, which resulted in more accurate estimates of the genetic and maternal environmental effects. The magnitude of the maternal environmental component can have a considerable effect on the heritability estimate. In the current study, maternal environmental effects explained 3% (SE = 1%) of the phenotypic variation in IgM versus 6% (SE = 2%) in the study by Berghof et al. [14]. When maternal environmental genetic effects were removed from the model, Berghof et al. [14] reported a heritability estimate of 0.29 for IgM. When maternal environmental effects in the current study were fixed at 6%, the heritability estimate for IgM reduced from 0.30 to 0.28.

Our analyses did not show evidence for maternal genetic effects on IgG and IgTotal, in spite of the large number of records included and collected over multiple generations. The standard error for estimates of the genetic correlation between direct and maternal genetic effects suggests that there is little information in the data to estimate this parameter when the model also includes a maternal environmental effect (Model (4)). For IgM, a model with a maternal genetic effect did result in a significantly better fit (p = 0.04) than a model with only a maternal environmental effect. However, the segregation of a gene with a major effect on IgM and allele frequencies that change during the course of selection, violates the assumption of equal genetic variance. As a result, a model with more degrees of freedom, as for the model that includes maternal genetic effects, could result in a better fit, even in the absence of true maternal genetic effects.

### Correlations

Estimates of genetic and phenotypic correlations reported in the current study are similar to those estimated by Berghof et al. [14] for the base population. The strong genetic correlations between of IgTotal with IgM and IgG can be explained by the part-whole relationship between these NAb. A stronger phenotypic correlation of IgTotal with IgG (0.84) than with IgM (0.54) reflects that the IgG in plasma are a larger contributor to IgTotal than IgM. The low environmental correlation between IgM and IgG (0.21) indicates that these two NAb titers are largely affected by different environmental factors. Production of antibodies in chicken includes two distinct processes: (1) primary production of IgM B cells, and (2) mechanisms that regulate class-switching of IgM to IgG or IgA (e.g. [20]). The process of class-switching is enhanced by environmental factors, usually antigenic challenges. Our results indicate that the production of IgM is under stronger genetic control than the process of class-switching to IgG. Our results also show that the environmental factors that affect the production of IgM differ largely from those involved in class-switching.

### Sex differences in NAb titers

In the study by Berghof et al. [14], the effects of plate and sex were confounded and therefore differences in NAb titers between females and males could not be estimated. From generation 1 onwards, the samples from males and females were analysed on the same ELISA plates and, therefore, the effects of sex and plate could be disentangled in the current study. In the base population, the blood plasma samples were taken at 15 weeks of age for the males and at 19 weeks of age for the females, but in the other generations, the blood plasma samples were taken at 16 weeks of age for both sexes. In generations 5 and 6, NAb titers were measured at both 16 and 32 weeks of age. Estimates of the genetic correlation between NAb titers for the same isotype (IgTotal, IgG or IgM) at these two ages were higher than 0.92 and not significantly different from 1 (results not shown). Therefore, it is unlikely that age differences between males and females in the base population affected results presented in Table 4.

It is generally accepted that males and females differ in their immune response, with females tending to mount stronger innate and adaptive immune responses than males, which can be due to the actions of genes, possibly due hormonal differences between males and females [21]. In the current study, the differences between NAb titers in males and females were small; males had 0.13 points higher IgM NAb titers than females. Furthermore, we detected a significant (*TLR1A* $\times$ Sex) interaction for IgM NAb titers; the *TLR1A* effect was larger in males than in females, which is consistent with the larger additive genetic variance for IgM in males than in females (Table 4). This scaling of *TLR1A* effects affects genetic variances but not the genetic correlation of IgM NAb titers between males and females (0.96). The estimate of the genetic correlation for IgTotal between males and females differed from 1 (0.78). This indicates that the effects of genes affecting IgTotal NAb titers differ between males and females. Sexual dimorphism has been described for e.g. body weight in broilers, with genetic correlation estimates ranging from 0.89 to 0.94, depending on age [22]. However, the genetic differences in immune response parameters between males and females have been scarcely analysed (e.g. [23]).

Previously, Berghof et al. [14] suggested that the maternal environmental effects for IgM might differ between females and males but it was not confirmed in the current study; the contribution of maternal environmental effects was very similar for males and females and estimates of the correlation between maternal environmental effects did not differ from 1.

### Selection strategy

The selection experiment described in the current study was designed based on the work of Wijga et al. [12] who estimated a heritability of 0.23 in layers for plasma NAb levels binding rabbit red blood cells based on agglutination assays. In the current study, the heritability estimate for the selection criterion, i.e. NAb for IgTotal binding KLH as measured by the much more sensitive ELISA technique, appeared to be considerably lower, with an estimate of 0.12 in the base population [14]. Therefore, selection methods other than mass selection might have been more efficient. The lower than expected estimate of heritability for this trait in the current population became available only after the experiment had started and we decided to continue with mass selection. Model calculations showed that alternative selection criteria, such as best linear unbiased predictions, would have resulted in an unacceptable increase in inbreeding, if not properly controlled. Furthermore, more complex breeding strategies would affect the logistics of the selection experiment. Thus, instead of changing the selection method, we decided to maximize the number of selection candidates within the restrictions set by the experimental facilities, and to reduce the generation interval. This allowed us to realize a considerable genetic difference between the lines while limiting the increase in inbreeding (see Additional file 3: Fig. S3), and thus the effect of random drift. During the course of the selection experiment, information on the segregation of a gene with a major effect on NAb in the base population became available [15] but this information was not included when selecting parents.

#### Selection response

The phenotypic trend shows that IgTotal titers in the Low line changed from 6.89 in the base population to 3.32 in generation 7 (Table 1). The High line did not show a similar increase in antibody titers, i.e. average IgTotal titers decreased from 6.89 in the base population to 5.96 in generation 7. This might lead to the incorrect conclusion that IgTotal titers can only be reduced and not increased by means of selective breeding. The estimated genetic trends differ in that respect from the phenotypic trends. For IgTotal, estimated genetic trends were very similar in the High and Low lines: genetic level in generation 7 was + 1.20 points (2.55σ_A_) in the High line and − 1.19 points (2.59σ_A_) in the Low line. Furthermore, the phenotypic difference in generation 7 was similar to the genetic difference; phenotypic and genetic differences between the High and Low lines for IgTotal were 2.64 (Table 1) and 2.39 titer points, respectively. The apparent discrepancy between the phenotypic and genetic trends (Table 1 vs Fig. 1) is due to the effects of ELISA plate (results not shown), which include generation (or batch) effects, i.e. specific environmental conditions during hatching and rearing of the chickens. Plate effects might also be due to factors such as laboratory conditions and reagent lots that cannot be overcome by using highly experienced and trained personnel or an internal standard, as in the current study. Moreover, we used one lot of KLH for coating and only two pooled reference samples for all ELISA tests during the whole selection experiment. Plate effects can, however, be accounted for in the statistical analysis, when using the appropriate experimental design, removing them from the estimates of genetic trends. In the current study, the samples from the two lines were analysed on the same plate, which allowed separation of the effects of line and plate. These plate or batch effects are a problem for several high-throughput technologies used in biology and, when not properly accounted for, can lead to incorrect conclusions [24], as demonstrated here when comparing the phenotypic and genetic trends.

Titer points are expressed on a log_2_-scale, and thus the High line had a 5.2 times higher IgTotal titer than the Low line. Comparing expected and realized selection responses for IgTotal shows that they closely agree for the Low line but response in the High line was lower than expected (see Additional file 3: Fig. S4). The expected selection response for IgTotal was based on standardized realized selection differentials (see Additional file 2: Figs. S1 and S2) and estimated genetic parameters (Table 2). These differences can be explained by allele frequency changes for the *TLR1A* polymorphism, which affect the genetic variance (see Additional file 4: Fig. S7) and were not accounted for when calculating expected selection responses. Theoretically expected changes in *TLR1A* allele frequencies due to mass selection for IgTotal were approximated using Falconer and Mackay [25] and were in line with observed allele frequency changes. In generation 7 of the High line, expected and observed *TLR1A C* frequencies were 0.77 and 0.66, respectively. For the Low line, expected and observed *TLR1A C* frequencies were 0.04 and 0.07, respectively (see Additional file 5: Fig. S8). When allele frequencies change as expected based on theory, the genetic variance due to *TLR1A* monotonically decreases in the High line from generation 1 to 7 (see Additional file 4: Fig. S7). However, in the Low line the genetic variation due to *TLR1A* first increases when the allele frequency changes from 0.45 to 0.23 and subsequently decreases. Allele frequencies might not only change due to selection but also due to drift. However, the increase in inbreeding per generation was low, i.e. on average 0.26% in the Low line and 0.31% in the High line (see Additional file 3: Fig. S3) and, therefore, allele frequency changes due to drift are expected to be small.

#### Correlated responses

Selection for IgTotal resulted in correlated responses for IgM and IgG. The difference in the magnitude of the direct and correlated selection responses is due to the genetic correlation between the selection criterion and the correlated trait and the relative size of the additive genetic standard deviation of the correlated trait (see Additional file 3: Fig. S5 and S6). The genetic trend for IgM clearly differed between the High and the Low selection lines and was not symmetrical: the genetic level in generation 7 in the Low line was -1.60 titer points (− 2.71σ_A_) in the Low line and + 1.13 titer points (+ 1.92σ_A_) in the High line. Asymmetric selection responses have been observed in several divergent selection experiments and have several possible explanations [25]. The asymmetrical selection response observed for IgM in the current study can be attributed to segregation of the *TLR1A* polymorphism with a full dominance mode of gene action. By generation 7, the response that was due to changes in allele frequency at the *TLR1A* gene was -0.65 titer points in the Low line and + 0.21 titer points in the High line. Selection response due to genes other than *TLR1A* was very similar for the High (− 0.95 titer points) and Low lines (+ 0.92 titer points).

In addition to the correlated responses in KLH-binding IgM and IgG, we have previously reported positive phenotypic correlations between NAb and natural auto-antibody isotypes that bind different antigens at different ages [26–28], and for total antibody isotype concentrations at 16 weeks of age [15]. Positive phenotypic correlations have also been reported for specific antibody responses, although they were antigen-dependent [27]. Moreover, chickens from the High line from generations 4 and 6 had improved avian pathogenic *Escherichia coli* (APEC) resistance compared to Low line chickens [11]. In addition, in generation 6, peripheral B-cell concentrations were higher in a subset of the High line females than in the Low line females. Relative bursa and spleen weights at 15 days of age were also larger in High line chickens than in Low line chickens of generations 4 and 6 [28]. These results collectively suggest that selection for higher KLH-binding NAb at 16 weeks of age improved humoral immune development and humoral immunity, which may improve the response of chickens to some diseases.

#### Variance

For all three NAb titers, their standard deviation was consistently higher in the Low line than in the High line, and this difference tended to increase with generation (Table 1). The same differences can be observed when studying residuals of the statistical models (results not shown). This differs from the increasing variance with the mean (scale effects that are commonly observed), which is expected to result in a higher variance in the High than in the Low line [23, 29]. Changes in allele frequencies at the *TLR1A* gene as a result of selection will affect the variance explained by this major gene (see Additional file 4: Fig. S7], which is not accounted for by Model (1). However, these changes in allele frequency can only explain about 5% of the observed changes in the phenotypic variation of IgTotal. Furthermore, the variation due to *TLR1A* in generation 7 was similar in the High and Low lines (see Additional file 4: Fig. S7). Therefore, changes in *TLR1A* allele frequencies cannot explain the observed differences in variation between the Low and High lines. More variation in the Low line could also point at increased environmental sensitivity in animals selected for low NAb levels [30].

### Effects of TLR1A

Based on a GWAS in the base population, we identified a genomic region on chromosome 4 with a major effect on IgM NAb [15]. Based on variant effect prediction tools, we identified the polymorphism in the *TLR1A* gene as the most likely candidate gene. Allele frequencies in the base population were derived based on imputed genotypes [15]. Chickens in generations 5, 6 and 7 were genotyped for this polymorphism. Using these data, we were able to confirm and further characterize the highly significant effects of *TLR1A* on IgTotal and IgM reported by Berghof et al. [15]. However, the estimated genotypic effects of *TLR1A* on IgM and IgTotal were about 69 and 19% larger, respectively, than those reported by Berghof et al. [15]. Berghof et al. [15] also reported a significant effect of *TLR1A* on IgG (p = 0.05), which was confirmed in the current study but with different estimates; the heterozygous advantage as suggested in the current study was not found by Berghof et al. [15].

The current data allowed us to test for the interaction of *TLR1A* with sex, line, and generation. Interactions of the *TLR1A* polymorphism with background genes might result in an interaction between *TLR1A* and line. The (*TLR1A* $\times$ Line) interaction on IgM was suggestive (p = 0.051) and the effects tended to be larger in the Low than in the High line. This is in line with the general observation that there was more variation in the Low than in the High line. Surprisingly, we detected highly significant *TLR1A* $\times$ generation interactions for IgM and IgTotal. The difference between the *CC*/*CG* and *GG* genotype classes for IgM was 0.70 titer points in generation 5, 1.22 titer points in generation 6, and 1.28 titer points in generation 7. Berghof et al. [15] reported a difference of 0.65 titer points between CC and GG genotypes in the base population for chickens kept on the facilities of Hendrix Genetics. In the selection experiment, the conditions were kept as stable as possible, but in spite of this standardization, generation-specific variation was present. Each generation of chickens was raised as a single batch and therefore several factors might be responsible for the observed *TLR1A* $\times$ generation interactions. It has been reported that season can influence the expression of *toll-like receptor* (*TLR*) genes via changes in temperature (e.g. [31]) and possibly in relative humidity, as described in humans [32]. Nutritional composition can be a direct source of variation in the expression of *TLR* genes (e.g. [33, 34]) and can also induce changes in microbiota composition [35], which can in turn affect expression of *TLR* genes (e.g. [36, 37]). Stress (corticosterone supplementation) was shown to influence the expression of *TLR* genes in the chicken bursa [38]. Therefore, feather pecking, cleaning, and changes in care takers cannot be excluded as potential factors that affect the effects of the *TLR1A* polymorphism in the different generations. Although the research facility has a high sanitary status, immune status and presence of pathogens could be another major factor that may have affected the expression of *TLR* genes in different generations (e.g. [39, 40]). Although the exact source of the observed differences in the effects of the *TLR1A* polymorphism between generations cannot be pinpointed, some results in the literature suggest a high sensitivity of the *TLR1A* polymorphism to environmental conditions. To the best of our knowledge, such strong genotype $\times$ environment interaction effects have not been reported before for traits related to disease resistance, either because such effects were not present or were not investigated.

We hypothesized that NAb play an important role in the immune defence of poultry. This seems to be in contradiction with the identification of an allele with a major deleterious effect on IgM Nab, since purifying (natural) selection is expected to remove alleles with deleterious effects from the population. However, pure line individuals are mostly kept under strict hygienic conditions in breeding nuclei, and therefore their immune response is not seriously challenged and purifying (natural) selection will not be effective. Furthermore, results from the selection experiment show that mass selection of pure line individuals for increased NAb levels will not be able to completely remove the *G* allele because of dominance of the *C* allele (see Additional file 5: Fig. S8).

### Implications

The reported *TLR1A* $\times$ generation interaction highlights the importance of test environment when selecting for KLH-binding NAb, and possibly for disease resistance-related traits in general. Strict hygienic conditions in commercial breeding nuclei might not seriously challenge the immune response and purifying (natural) selection for alleles with detrimental effects on immune response might not be effective. For selection decisions, breeding companies not only rely on the performance of pure line individuals but also on the crossbred phenotypes from progeny that are kept under commercial conditions with less environmental control and that are therefore more challenging for the immune system. However, when the other parental line is homozygous for the favourable *TLR1A* allele (*CC*), differences in the performance of crossbred offspring will not be affected by the *TLR1A* polymorphism because of the dominant gene action. Therefore, we hypothesize that, in crossbreeding schemes, most of the alleles with detrimental effects on immune response remain undetected and that this may lead to the accumulation of deleterious mutations in elite purebred lines. This might not be limited to genes related to disease resistance but might also apply to other genes if genotype-by-environment interaction plays a role and mutations show dominant gene action. One might argue that this is not a problem since it does not affect the performance of commercial crossbred offspring. However, in the long term, accumulation of alleles with deleterious effects on disease resistance in the pure lines will gradually increase the risk of disease outbreaks in the pure lines. Such a disease outbreak can have far-reaching consequences for commercial breeding companies. Recently, it has been shown that deleterious alleles can be identified based on whole-genome sequence analyses (e.g. [41]). Therefore, sequencing pure line individuals might offer opportunities to select against these deleterious mutations. Alternatively, breeding companies might test pure line animals under commercial conditions.

## Conclusions

We successfully selected chickens for high and low NAb titers. Results confirm a major effect of a polymorphism in the *TLR1A* gene on IgTotal and IgM NAb and provide experimental verification of changes in allele frequencies at a major gene with dominant gene action on a quantitative trait that is subjected to mass selection. The observed asymmetrical selection response could be attributed to segregation of the *TLR1A* polymorphism. *TLR1A* by generation interactions suggest high sensitivity to environmental factors and illustrate the importance of choosing the appropriate environment when selecting for NAb and, possibly, for disease resistance in general. Results from this study suggest that in crossbreeding systems, with pure-line animals kept under strict hygienic conditions, there is no purifying selection against alleles with detrimental effects on disease resistance and a dominant mode of gene action. Therefore, alleles with detrimental effects on disease resistance might accumulate in pure line animals.

## Supplementary Information

**Additional file 1: Table S1.** Number of selected sires and dams for the High and Low selection lines.

**Additional file 2: Figure S1.** Standardized realized selection differentials for males in the High and Low selection lines. **Figure S2.** Standardized realized selection differentials for females in the High and Low selection lines.

**Additional file 3: Figure S3.** Average inbreeding in the High and Low selection lines. **Figure S4.** Realized and expected selection response for IgTotal in the High and Low selection lines. **Figure S5.** Realized and expected correlated selection response for IgM in the High and Low selection lines. **Figure S6.** Realized and expected correlated selection response for IgG in the High and Low selection lines.

**Additional file 4: Figure S7.** Additive genetic variance due to the *TLR1A* polymorphism as a function of the *C* allele frequency. Frequency of the *TLR1A C* allele (based on imputed genotypes) was 0.45 in the base population [15], 0.04 and in generation 7 of the Low line and 0.66 in generation 7 of the High line.

**Additional file 5: Figure S8.** Realized and expected changes of *TLR1A* allele frequencies. Theoretically expected changes in *TLR1A* frequencies due to mass selection for IgTotal.

## Data Availability

The data analysed in this study are not publicly available as part of the data is owned by Hendrix Genetics (Boxmeer, The Netherlands).
