# Supplementary material for: Divergent selection for natural antibodies in poultry in the presence of a major gene
Source: Genet Sel Evol. 2022 Mar 21;54:24. doi: 10.1186/s12711-022-00715-9 (PMC8939063; doi:10.1186/s12711-022-00715-9)
Supplement: Supplementary file 1 — Additional file 1: Table S1. Number of selected sires and dams for the High and Low selection lines. [file 12711_2022_715_MOESM1_ESM.docx]

**Additional file 1 Table S1**

In each generation, we aimed at selecting 25 males and 50 females. The actual number of selected sires and dams are in Table 1.

Table S1. Number of selected sires and dams for the High and Low selection lines

|  | High line | |  | Low line | |
| --- | --- | --- | --- | --- | --- |
|  | Sire | Dam |  | Sire | Dam |
| G0 | 24 | 44 |  | 24 | 48 |
| G1 | 25 | 50 |  | 25 | 50 |
| G2 | 25 | 50 |  | 25 | 49 |
| G3 | 25 | 49 |  | 25 | 50 |
| G4 | 25 | 50 |  | 25 | 50 |
| G5 | 25 | 50 |  | 25 | 48 |
| G6 | 22 | 44 |  | 23 | 39 |
