# Supplementary material for: Divergent selection for natural antibodies in poultry in the presence of a major gene
Source: Genet Sel Evol. 2022 Mar 21;54:24. doi: 10.1186/s12711-022-00715-9 (PMC8939063; doi:10.1186/s12711-022-00715-9)
Supplement: Supplementary file 2 — Additional file 2: Figure S1. Standardized realized selection differentials for males in the High and Low selection lines. Figure S2. Standardized realized selection differentials for females in the High and Low selection lines. [file 12711_2022_715_MOESM2_ESM.docx]

**Additional file 2 Figures S1 and S2**


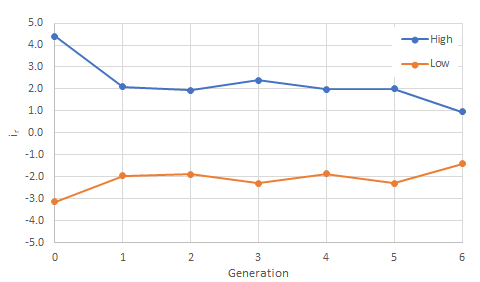


Figure S1. Standardized realized selection differentials for males in the High and Low selection line.


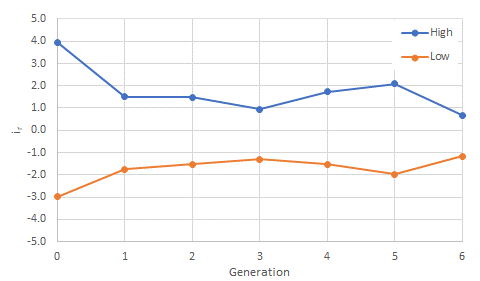


Figure S2. Standardized realized selection differentials for females in the High and Low selection lines.

Standardized realized selection differentials (i_r_) were calculated for each line by generation combination as:

$i_{r}= \frac{\mu_{Sel}-\mu_{Pop}}{\sigma_{p}}$,

where $\mu_{Sel}$ is the mean NAb IgTotal titer of the selected males/females, $\mu_{Pop}$ is the mean NAb IgTotal titer of all male/female selection candidates and $\sigma_{p}$ is the phenotypic standard deviation for NAb IgTotal titer, estimated based on all male/female selection candidates.
