# Supplementary material for: Divergent selection for natural antibodies in poultry in the presence of a major gene
Source: Genet Sel Evol. 2022 Mar 21;54:24. doi: 10.1186/s12711-022-00715-9 (PMC8939063; doi:10.1186/s12711-022-00715-9)
Supplement: Supplementary file 3 — Additional file 3: Figure S3. Average inbreeding in the High and Low selection lines. Figure S4. Realized and expected selection response for IgTotal in the High and Low selection lines. Figure S5. Realized and expected correlated selection response for IgM in the High and Low selection lines. Figure S6. Realized and expected correlated selection response for IgG in the High and Low selection lines. [file 12711_2022_715_MOESM3_ESM.docx]

**Additional file 3 Figures S3 to S6**

**Realized and expected (correlated) selection response**

**Inbreeding**


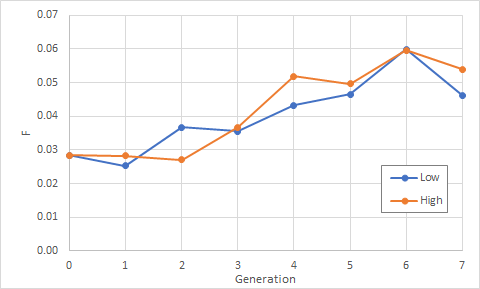


Figure S3. Average inbreeding in the High and Low selection lines

**IgTotal**

Theoretically expected selection response for IgTotal was calculated as

$\Delta_{t}=\frac{i_{r_{m}}\cdot h\cdot\sigma_{A}+i_{r_{f}}\cdot h\cdot\sigma_{A}}{2}$,

where $i_{r_{m}}$ and $i_{r_{f}}$ are the standardized realized selection differentials for males and females, respectively, in a specific generation, $h$ is the square root of the heritability for IgTotal and $\sigma_{A}$ is the additive genetic standard deviation for IgTotal.

Standardized realized selection differentials for males and females are in Additional file 2. The heritability was 0.12 and the additive genetic standard deviation 0.471 (see Table 2)

The genetic level in subsequent generations was calculated as:

$\bar{A_{t+1}}=\bar{A_{t}}+\Delta_{t}$.

Theoretically expected responses are approximations which do not account for e.g. unequal contributions of selected parents to the next generation, change in additive genetic variance due to changes in *TLR1A* allele frequencies and the Bulmer effect. Furthermore, the heritability used was based on a model that adjusted for Plate effects whereas (mass) selection was based on unadjusted IgTotal titers.


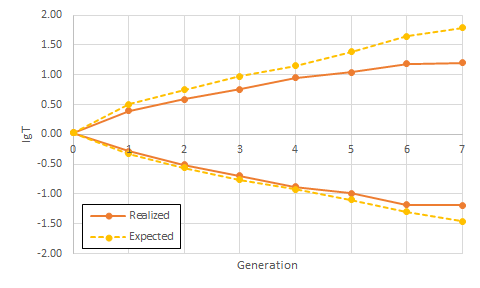


Figure S4. Realized and expected selection response for IgTotal in the High and Low selection lines

**IgM**

Theoretically expected correlated changes in IgM due to selection for IgTotal were calculated

$CR=i_{r}\cdot r_{a}\cdot h_{IgTotal}\cdot\sigma_{A-IgM}$,

where $i_{r}$ is the standardized realized selection differentials, $r_{a}$ is the genetic correlation between IgTotal and IgM (0.91), $h_{IgTotal}$ is the square root of the heritability for IgTotal and $\sigma_{A-IgM}$ is the additive genetic standard deviation for IgM (0.587). Parameters were taken from Table 2 and Table 3. Other calculations are as described for IgTotal.


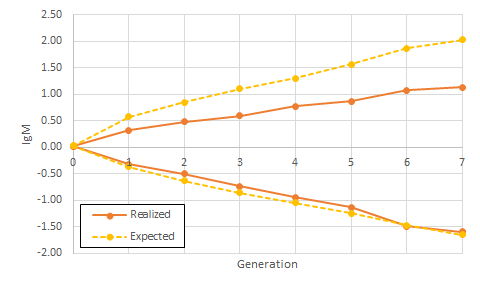


Figure S5. Realized and expected correlated selection response for IgM in the High and Low selection lines

**IgG**

Theoretically expected correlated changes in IgM due to selection for IgTotal were calculated

$CR=i_{r}\cdot r_{a}\cdot h_{IgTotal}\cdot\sigma_{A-IgG}$,

where $i_{r}$ is the standardized realized selection differentials, $r_{a}$ is the genetic correlation between IgTotal and IgG (0.94),$h_{IgTotal}$ is the square root of the heritability for IgTotal and $\sigma_{A-IgG}$ is the additive genetic standard deviation for IgG (0.484). Parameters were taken from Table 2 and Table 3. Other calculations are as described for IgTotal.


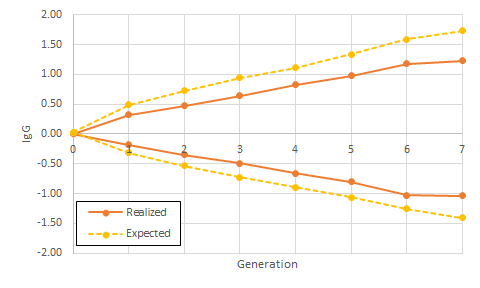


Figure S6. Realized and expected correlated selection response for IgG in the High and Low selection lines
