# Supplementary material for: Divergent selection for natural antibodies in poultry in the presence of a major gene
Source: Genet Sel Evol. 2022 Mar 21;54:24. doi: 10.1186/s12711-022-00715-9 (PMC8939063; doi:10.1186/s12711-022-00715-9)
Supplement: Supplementary file 4 — Additional file 4: Figure S7. Additive genetic variance due to the TLR1A polymorphism as a function of the C allele frequency. Frequency of the TLR1A C allele (based on imputed genotypes) was 0.45 in the base population [15], 0.04 and in generation 7 of the Low line and 0.66 in generation 7 of the High line. [file 12711_2022_715_MOESM4_ESM.docx]

**Additional file 4 Figure S7**

**Additive genetic variance due to the TLR1A polymorphism**

The additive genetic variance due to the TLR1A polymorphism was calculated as:

$V_{QTL}=2pq\left[ a+\left( 1-2p \right)d \right]^{2}$,

where $p$ is the frequency or the *TLR1A C* allele, $a$=0.22 and $d$=0.27 are based on estimated genotypic effects for IgTotal (see Table 5)


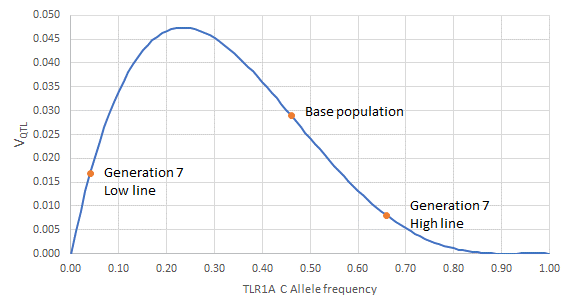


Figure S7. Additive genetic variance due to the *TLR1A* polymorphism as a function of the *C* allele frequency. Frequency of the *TLR1A C* allele (based on imputed genotypes) was 0.45 in the base population [15], 0.04 and in generation 7 of the Low line and 0.66 in generation 7 of the High line.
