# Supplementary material for: Divergent selection for natural antibodies in poultry in the presence of a major gene
Source: Genet Sel Evol. 2022 Mar 21;54:24. doi: 10.1186/s12711-022-00715-9 (PMC8939063; doi:10.1186/s12711-022-00715-9)
Supplement: Supplementary file 5 — Additional file 5: Figure S8. Realized and expected changes of TLR1A allele frequencies. Theoretically expected changes in TLR1A frequencies due to mass selection for IgTotal. [file 12711_2022_715_MOESM5_ESM.docx]

**Additional file 5 Figure S8**

**Realized and expected changes of *TLR1A* allele frequencies**

Theoretically expected changes in *TLR1A* frequencies due to mass selection for IgTotal were approximated using Falconer and Mackay [25]. The coefficient of selection was approximated as:

$s=i_{r}\left( \frac{2a}{\sigma_{p}} \right)$,

where $i_{r}$ is the standardized realized selection differentials (see Additional file 2 Figures S1 and S2), $a$ is the additive genetic effect of the *TLR1A* polymorphism on IgTotal ($a$=0.22; see Table 5) and $\sigma_{p}$ is the phenotypic standard deviation of IgTotal ($\sigma_{p}$=1.36; see Table 2).

Imputed genotypes for the *TLR1A* SNP were available for the base population [15].The change in *TLR1A G* frequency was calculated as in [25]:

$\Delta q=-sq^{2}\left( 1-q \right)$.

Changes in *TLR1A G* frequency were calculated separately for males and females and subsequently averaged.

Calculations assume that *TLR1A* effects are constant over generations and show complete dominance i.e. assuming that effects of *CC* and *GG* on IgTotal are identical. Furthermore, the phenotypic standard deviation ($\sigma_{p}$) is from a model that accounts for plate effects whereas (mass) selection was based on unadjusted IgTotal titers.


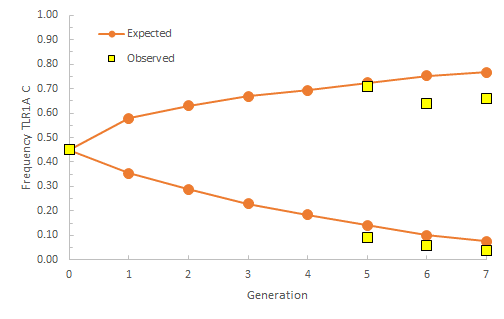


Figure S8. Realized and theoretically expected changes of *TLR1A C* allele frequencies.
